# All-solid-state lithium-oxygen battery with high safety in wide ambient temperature range

**DOI:** 10.1038/srep13271

**Published:** 2015-08-21

**Authors:** Hirokazu Kitaura, Haoshen Zhou

**Affiliations:** 1 Energy Technology Research Institute, National Institute of Advanced Industrial Science and Technology, Umezono, 1-1-1, Tsukuba, 305-8568, JAPAN

## Abstract

There is need to develop high energy storage devices with high safety to satisfy the growing industrial demands. Here, we show the potential to realize such batteries by assembling a lithium-oxygen cell using an inorganic solid electrolyte without any flammable liquid or polymer materials. The lithium-oxygen battery using Li_1.575_Al_0.5_Ge_1.5_(PO_4_)_3_ solid electrolyte was examined in the pure oxygen atmosphere from room temperature to 120 °C. The cell works at room temperature and first full discharge capacity of 1420 mAh g^−1^ at 10 mA g^−1^ (based on the mass of carbon material in the air electrode) was obtained. The charge curve started from 3.0 V, and that the majority of it lay below 4.2 V. The cell also safely works at high temperature over 80 °C with the improved battery performance. Furthermore, fundamental data of the electrochemical performance, such as cyclic voltammogram, cycle performance and rate performance was obtained and this work demonstrated the potential of the all-solid-state lithium-oxygen battery for wide temperature application as a first step.

The lithium-oxygen battery has attracted much attention due to its high theoretical energy density of 3500 Wh kg^−1^. This energy density comes from the electrochemical reaction of two low-mass elements, lithium and oxygen. It is estimated that between 300–1000 Wh kg^−1^ (based on package) of actual energy density is achievable^1^,^2^,^3^. In order to develop the Li-O_2_ batteries, highly-stable electrolytes are required because activated oxygen attacks unstable electrolytes and they are easily decomposed. It follows that the target reaction between lithium and oxygen is inhibited^4^,^5^,^6^,^7^. Therefore, carbonate-based organic liquid electrolytes commonly used in lithium ion batteries cannot be used in the Li-O_2_ battery and many researchers have investigated materials for use as stable electrolytes. Recently, some relatively stable organic electrolytes compared with the carbonate-based electrolytes have been reported. Jung *et al*. reported that the cell using glyme electrolytes show stable discharge-charge performance and that a theoretical reaction (2Li^+^ + O_2_ + 2e^−^ ⇄ Li_2_O_2_) proceeded^8^. 1,2-dimethoxyethane (DME) also has been used^7^. Very recently, Adams *et al*. demonstrated 2,3-dimethyl-2,3-dimethoxybutane (DMDMB) shows the higher stability and a lot of efforts are being made to search much more stable electrolytes^9^.

Inorganic solid lithium ion conductors have been studied as solid electrolytes for lithium ion batteries and it has been proved that they show the high electrochemical stability and long cycle life^10^,^11^,^12^,^13^,^14^,^15^,^16^. Hence, it is considered that the solid materials are one of the candidates as electrolytes for Li-O_2_ batteries. Additionally, the application of solid electrolytes to the Li-O_2_ batteries can enhance the safety owing to their flame resistant properties. The high thermal stability of solid electrolytes prevents the thermal runaway of the batteries and concurrently allows them to operate effectively at higher temperature. Higher temperature operation is beneficial to improve the cell performance because the lithium ion conductivity is increased and the catalytic oxygen reduction reaction (ORR) and oxygen evolution reaction (OER) at the air electrode are activated.

Kumar *et al*. firstly fabricated the solid-state Li-O_2_ battery using Li_y_Al_x_Ge_2*−*x_(PO_4_)_3_ (LAGP) as a solid electrolyte and polymer electrolytes as a buffer layer to decrease the contact resistance between LAGP and air electrode^17^. We have developed the all-solid-state type battery without any polymer materials to realize the room temperature operation because polymer electrolytes show low conductivity at room temperature. A sintered air electrode was used to decrease the contact resistance and Li/LAGP/LAGP-carbon nanotube (CNT) cell was fabricated. We first challenged to obtain the electrochemical performance of the cell in pure oxygen atmosphere. However, it was difficult to operate the cell because of immature fabrication technique during an earlier stage. Then we attempted the cell operation in ambient atmosphere and the cell can be discharged and charged. Now we consider the difference between failure in oxygen atmosphere and success in air is attributed to the moisture, which may play a role as an ionic conductor in the air electrode. Therefore, we have advanced the cell as a Li-“air” battery and reported the electrochemical performance of the cell in ambient air atmosphere^18^. As a result of efforts, the cell could be discharged and charged for 10 cycles. However, Li_2_CO_3_ and LiOH were produced by the chemical reaction between discharge products and CO_2_/H_2_O in air, and during charging, high voltage over 4 V was needed for the decomposition of these by-products to refresh the air electrode. This result indicated that the lithium-air battery should be discharged and charged in the condition without CO_2_/H_2_O to avoid the high voltage charging even if the lithium anode is protected from CO_2_/H_2_O by the solid electrolyte layer. Therefore, we had to go back to the starting point of the cell operation in the pure oxygen atmosphere.

In this report, we show the electrochemical performance at RT in the pure oxygen atmosphere on the Li/LAGP/LAGP-CNT cells constructed by the optimized conditions such as sintering condition and thickness of the air electrode. In addition, the cell performance at relatively high temperature is investigated to proof that this all-solid-state cell shows the high safety and can be used in the wide temperature range.

## Results

**Figure 1:**
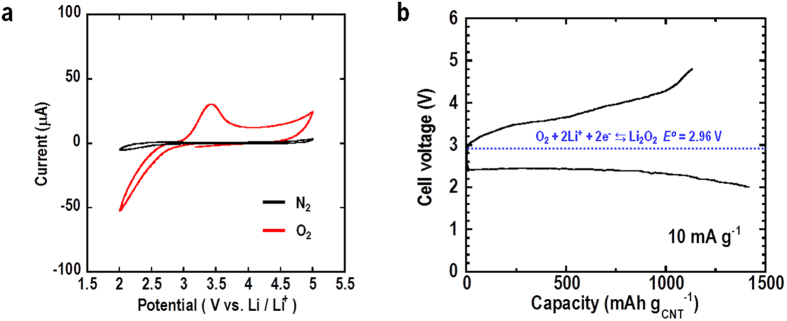
Cyclic voltammogram and discharge-charge curves for all-solid-state Li-O_2_ cell. (**a**) Cyclic voltammogram of cell at scan rate of 10 mV s^−1^ at room temperature in O_2_ and N_2_ atmosphere. (**b**) 1st discharge-charge curves for cell under constant current density of 10 mA g^−1^ in voltage range of 2.0–4.8 V at room temperature in an O_2_ atmosphere.

A bulk-type all-solid-state Li-O_2_ cell (Li/LAGP/LAGP-CNT) was fabricated by using sintering process similar to the previously-reported process^18^. The inert atmosphere during sintering process has been optimized to avoid the decomposition of CNT and the electrode thickness was fixed around 20 μm. In this case, the mass of air electrode was about 0.5 mg in the area of Φ = 6 mm. Firstly, the potential of the electrochemical reactions in the Li/LAGP/LAGP-CNT-O_2_ cell at RT was investigated using cyclic voltammetry (CV) and then the discharge-charge measurements were conducted as shown in Fig. 1. CV measurement was carried out in N_2_ and O_2_ atmosphere at a scan rate of 10 mV s^−1^. CV curve in O_2_ atmosphere showed a reduction current at a potential of less than 3 V, and an anodic peak between 3.1 and 4.0 V. Compared with CV curve in N_2_ atmosphere, it is considered that redox reactions are derived from the ORR and decomposition of products formed by ORR. Then the cell was discharged and charged using a constant current density of 10 mA g^−1^ in the voltage range of 2.0–4.8 V at RT (Fig. 1b). The current density and cell capacity were normalized by the weight of CNT calculated from the weight of air electrode after sintering and the mixing ratio of LAGP and CNT. The cell initially provided a discharge voltage of 2.4 V, which gradually decreased to 2.0 V as the discharge process continued. The discharge capacity was about 1420 mAh g^−1^, and a recharge capacity of about 1130 mAh g^−1^ could be obtained when the cell was charged up to 4.8 V. These values can be recalculated to discharge and charge capacity of 120 and 96 μAh cm^−2^, respectively, at the current density of 0.85 μA cm^−2^. This voltage profile in the constant current condition matches the result obtained by CV measurement. These redox potentials are consistent with the reported potentials for ORR and OER^2^,^8^,^19^.

**Figure 2:**
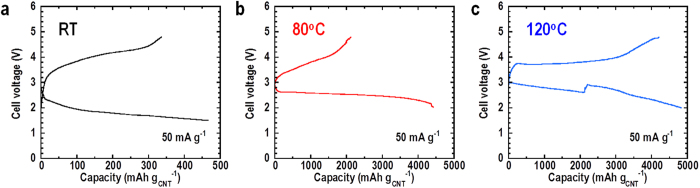
1st discharge-charge curves of all-solid-state Li-O_2_ cell under current density of 50 mA g^−1^ at different temperatures. (**a**) Discharge-charge curves for cell at room temperature in voltage range of 1.5–5.0 V. (**b**) Discharge-charge curves for cell at 80 °C in the voltage range of 2.0–4.8 V. (**c**) Discharge-charge curves for cell at 120 °C in the voltage range of 2.0–4.8 V.

The rate performance of this all-solid-state Li-O_2_ battery was also investigated. The current density was increased to 50 mA g^−1^ and the voltage region was extended to between 1.5–5.0 V (Fig. 2a). Although the overpotential increased, the cell could still be smoothly discharged and charged at this current density. A discharge capacity of 460 mAh g^−1^ was obtained at RT. Then, the cell performance was investigated by controlling the current density at each step to understand the discharge-charge behavior at different current densities (Figure S1a and S1b). The results showed that the cell can be initially discharged/charged in the range 2.0–4.0 V at the current densities less than or equal to 20 mA g^−1^, and that an extended voltage region was apparent at the current densities over 20 mA g^−1^. In addition, the rate performance can be enhanced by reducing the thickness of the solid electrolyte layer. The thickness was changed from 1 mm (Figure S1a and S1b) to 0.5 mm (Figure S2a and S2b). The rate performance improved significantly; for example, the discharge and charge voltages at 200 mA g^−1^ were in the range 2.0–4.0 V. These results indicate that the resistance of solid electrolytes is still a rate limiting factor. Therefore, improvement of lithium-ion conductivity and decreasing the thickness of the solid electrolyte layer will enhance cell performance and bring it closer to becoming a practical battery.

**Figure 3:**
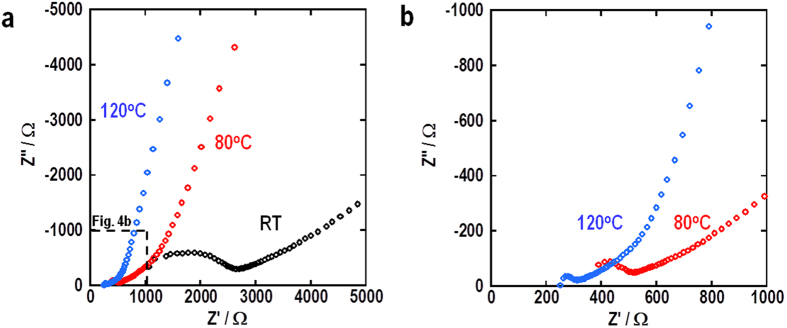
Impedance spectra for all-solid-state Li-O_2_ cell at different temperatures before discharging. (**a**) Impedance spectra and (**b**) enlarged view of part of data at room temperature, 80 °C, and 120 °C. Black, red and blue circles indicate the spectra at room temperature, 80 °C and 120 °C, respectively.

Another way to enhance the cell performance is to increase the temperature, since the lithium ion conductivity and reaction kinetics are enhanced at increased temperature. In addition, having a wide operating temperature range is an advantage since the battery can be used in various environments. Figure 3a shows the impedance spectra of the cells at RT, 80 °C and 120 °C; Fig. 3b shows an enlarged view of part of Fig. 3a. The data described a semicircle in the high frequency region and a linear trend in the low frequency region. The total cell resistance was determined by the intersection of the semicircle at the low frequency side. The cell at RT showed a resistance of about 2600 Ω, which decreased with increasing temperature. The cell resistance was about 500 Ω and 300 Ω at 80 °C and 120 °C, respectively. The unchanged shape of the impedance spectra at elevated temperature indicated that heating caused no unfavorable, severe reactions between the electrode materials.

The discharge and charge curves for the cells at 80 °C and 120 °C are shown in Fig. 2b,c, respectively. Even at a current density of 50 mA g^−1^, large discharge capacities of over 4000 mAh g^−1^ were obtained for both cells. The overpotential at the discharge process in the cell at 80 °C decreased compared with the cell at RT. The results shown in Figure S3a and S3b also demonstrated the enhancement of rate performance at 80 °C. The discharge and charge voltages at 2000 mA g^−1^ were still in the range 2.0–4.0 V. The cell exhibited not only increased discharge capacity, but also enhanced reversibility when the temperature was elevated further to 80 °C and 120 °C. Park *et al*. showed that a Li-O_2_ cell using TEGDME-LiCF_3_SO_3_ can be operated in a wide temperature range of −10–70 °C^20^. Our results demonstrated that the application of solid electrolytes to Li-O_2_ cells extends the operation temperature and allows safe operation at over 80 °C. The flat charge curve at 120 °C is different from the sloped curve obtained at RT and 80 °C, and the increasing discharge voltage in the middle would indicate the incorporation of air due to the thermal expansion of the sealed container; we had previously observed a flat charge profile in the cell when operated in an open air atmosphere^18^. Therefore, development of a cell specially designed to operate at elevated temperatures is required for the further high-temperature experiments.

**Figure 4:**
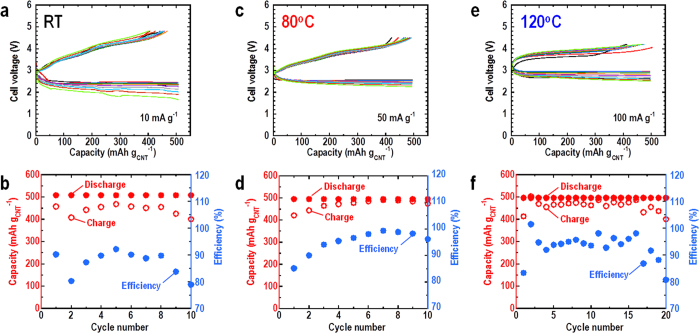
Cycle performance of all-solid-state Li-O_2_ cell with capacity limit of 500 mAh g^−1^ at different temperatures. (**a**) 1st–10th discharge-charge curves and (**b**) cycle performance for cell at current density of 10 mA g^−1^ with the charge cut-off voltage of 4.8 V at room temperature. (**c**) 1^st^–10^th^ discharge-charge curves and (**d**) cycle performance for cell at current density of 50 mA g^−1^ with the charge cut-off voltage of 4.5 V at 80 °C. (**c**) 1st–20th discharge-charge curves and (**d**) cycle performance for cell at current density of 100 mA g^−1^ with the charge cut-off voltage of 4.2 V at 120 °C. Red, blue and black circles indicate the discharge capacity, charge capacity and capacity efficiency, respectively.

The cycle performance at each temperature is shown in Fig. 4. All the measurements were conducted with a capacity limit of 500 mAh g^−1^ in accordance with a commonly-used technique^8^,^20^,^21^,^22^. The charge cut-off voltage was set at the voltage after rise observed in Fig. 2. Figure 4a,b show the 1st–10th discharge-charge curves, and the cycle performance, respectively, using a current density of 10 mA g^−1^ with the charge cut-off voltage of 4.8 V at RT. The cell retained a discharge capacity of 500 mAh g^−1^ during 10 cycles. The discharge voltage decreased with the cycling. On the other hand, the charge voltage did not increase so much. Therefore, this degradation was not caused by the degradation of the interface between Li and LAGP. One possibility is the slightly low cycle efficiency of 80–90%, which caused gradual accumulation of discharge products, led to the decrease of ORR activity. This degradation can be mitigated by elevating the temperature to 80 °C, as shown in Fig. 4c,d. The test conditions were slightly different from the conditions at RT: the current density was 50 mA g^−1^ and the charge cut-off voltage was 4.5 V. The cell showed a high efficiency of 95–99% from the 4th cycle onwards, resulting in a slight degradation of the discharge voltage with cycling compared with the cell at RT. In the results shown in Fig. 4e,f, the cell was cycled at a higher temperature of 120 °C: the current density was 100 mA g^−1^ and the charge cut-off voltage was 4.2 V. Under these conditions, the cell also retained its discharge capacity during 20 cycles. The discharge-charge efficiency was 90–98% from the 3rd cycle to 16th cycle, then decreased. The lower efficiencies compared with that obtained at 80 °C would be caused by air incorporation due to the cell not being specially designed for the operation at high temperature.

## Discussion

To apply the solid electrolyte to Li-O_2_ battery, the features of high lithium ion conductivity, high atmospheric stability and high stability against lithium metal for solid electrolytes are needed. However, there are not many kinds of solid electrolytes to meet these needs. Li_7_La_3_Zr_2_O_12_ (LLZ) is expected as a most promising electrolyte, especially in terms of its high stability against lithium metal^23^,^24^. However, LLZ is suffering from the construction of solid-solid interface because its high sintering temperature leads to the lithium absence and formation of some impurity phases. Hence, LLZ needs more great efforts to fabricate the good solid-solid interface and will permit to be used for the solid-state batteries in the future. On the other hand, LAGP has the advantages that the sintering temperature is relatively low and the quantity synthesis is easy. However, recent progress showed that LAGP gradually reacts with Li metal hour by hour^25^. In that literature, it was reported that germanium was reduced from Ge^4+^ to Ge^3+^ and the impedance in the Li/LAGP/Li cell increases continuously with time. Actually, the cell using LAGP showed relatively large cell resistance of 2600 Ω at RT as shown in Fig. 3a. The cell resistance includes Li/LAGP interfacial, LAGP bulk, LAGP grain boundary, and LAGP-CNT cathode resistances.The resistances of LAGP layer (=LAGP bulk + LAGP grain boundary) and cathode can be roughly estimated by the lithium ion conductivity of LAGP and their thicknesses. Half of cell resistance is derived from the resistance of LAGP layer, and the cathode resistance corresponds to a few hundred Ω. The rest of the resistance of about 1000 Ω is attributed to the Li/LAGP interfacial resistance. The highest resistance in this cell is LAGP layer because thick LAGP pellet with the thickness of 1 mm was used for easier handling. This resistance can be reduced by using thiner LAGP layer. Li/LAGP interfacial resistance is also high. This resistance would be attributed to the formation of high resistance layer between Li and LAGP when the melting Li was contacted to LAGP. According to the literature, this resistance may increase with time. Therefore, the cell using LAGP may not endure the long-term operation and LAGP should be replaced with more favorable solid electrolytes such as LLZ to examine the higher rate performance and longer cyclability. We confirmed that this LAGP cell can work under low current densities for at least 1500 hours even if Li/LAGP interfacial resistance increases^18^. Therefore, fundamental data obtained in this problem-free period was demostrated in this paper.

From these results, the bulk-type all-solid-state Li-O_2_ cell was successfully operated at RT in the dry oxygen condition. To our knowledge, the construction of a bulk-type all-solid-state “lithium ion” battery using oxide solid electrolytes such as LAGP is difficult due to the difficulty in fabricating a suitable electrode-electrolyte interface arises from the high sintering temperature required to produce dense grain boundary, leading to an unfavorable reaction between the active materials and the solid electrolytes. Therefore, there have been few reports of the successful development of cells exhibiting adequate electrochemical performance in the lithium ion battery^26^,^27^. On the other hand, in the current all-solid-state Li-O_2_ cell, the active material is oxygen gas, and the components of the air electrode during the sintering process are only carbon and solid electrolyte materials. Thus, there is no unfavorable reaction during the sintering process for this electrode. Furthermore, the gas-solid interface can be readily fabricated to provide a good electrode-electrolyte interface for the electrochemical reaction sites. These two factors led to the success of the bulk-type all-solid-state Li-O_2_ cell using the oxide solid electrolyte described here.

It should be noted that the charge curve starts from 3.0 V, and that the majority of it lies below 4.2 V. This voltage profile under constant current conditions matches the results obtained by CV measurements. These redox potentials are consistent with the reported potentials for ORR and OER^2^,^8^,^19^. In particular, cells using relatively stable organic liquid electrolytes such as glyme, DME and DMDMB provide CV results similar to those reported here^8^,^9^,^20^,^28^. In addition, the all-solid-state cell repeatedly showed the same charge curve and a rise at the end of the charge curve, like other traditional rechargeable batteries, meaning that continuous electrochemical decomposition of electrolyte and electrode materials would not occur in this voltage range. Therefore, it is considered that the solid electrolyte in this all-solid-state Li-O_2_ cell is relatively stable. On the other hand, the charge curve showed the high voltage at the end of charging compared with the voltage at the beginning of charging. It would be caused by the overpotential attributable to the decomposability of discharge products. It is considered that the decomposition of the part of discharge products apart from the electrode is difficult compared with the part of discharge products close to the electrode and the overpotential gradually increases as charging progress. Hu *et al*. demonstrated the size effect of Li_2_O_2_ on charging performance^29^. In their results, smaller Li_2_O_2_ showed the low charge voltage. Therefore the generative morphology control of discharge products will become a key technology for decreasing the whole charge potential even in the all-solid-state Li-O_2_ battery.

Another key technology to improve the cell performance is electrode design. The specific capacity and current density were normalized by the mass of carbon as with many literatures. However, this cell contains a large amount of LAGP in the cathode. When calculated from the mass of cathode, the capacity corresponding to 1420 mAh g_carbon_^−1^ is about 70 mAh g_cathode_^−1^. Although the capacity based on the mass of carbon is not so small compared with the cells using CNT and liquid electrolytes, the capacity based on the mass of whole cathode is very small^30^,^31^. Therefore, the decreasing the ratio of LAGP within cathode is important. We have demonstrated that the ratio of solid electrolytes and CNT affects the cell performance^32^. The cell performance decreased with increasing in the percentage of CNT and 33% addition of CNT led to the very small capacity. Hence, the innovative design for the air electrode to reduce the percentage of LAGP is needed.

The electrochemical reaction mechanism involves the formation and dissociation of discharge products, which are lithium oxide compounds^2^. In cells using glyme electrolytes, the main discharge product is Li_2_O_2_^8^. Although we attempted to determine the discharge products for all-solid-state Li-O_2_ cell using X-ray diffraction and Raman spectroscopy, we were unsuccessful due to the small amounts of product produced. Assuming that Li_2_O_2_ was produced on the surface of LAGP and CNT, the calculated thickness is about several nm after discharging to the capacity of 1420 mAh g^−1^. Therefore, we performed X-ray photoelectron spectroscopy (XPS) measurements as this is a highly-sensitive method that can detect small amounts of product materials. In XPS spectra (Figure S4), the intensity of the Li 1s peak for the air electrode after discharge increased and the peak shifted to lower energy, indicating that some compounds containing lithium existed. Based on the elements present and previous reports, the most likely candidates for the produced compounds are Li_2_O_2_, LiO_2_, Li_2_O and Li_2_CO_3_^33^,^34^. No change in the intensity around 290.0 eV, which is related to Li_2_CO_3_, was observed in the C1s spectrum in Figure. S5, which indicates that the produced lithium compounds are different from lithium carbonate. In addition, Li_2_O generally has a characteristic O1s peak around 528.6 eV, which was not observed^34^. LiO_2_ is metastable and eventually becomes Li_2_O_2_^33^. Hence, the observation of LiO_2_ would be difficult using this *ex-situ* measurement. Shao-Horn *et al*. demonstrated that Li_2_O_2_ is electrochemically produced on the surface of inorganic materials in a solid-state thin film air battery, and that the binding energy of Li 1s in this battery is about 54.7 eV^35^. In our results, Li 1s spectrum before discharge showed a broad peak with a maximum at around 55.2 eV, which is consistent with XPS results of LAGP^25^. The peak shifted to lower energy after discharge. Assuming that the peak actually consists of two peaks associated with LAGP and another lithium compound, the peak could be divided into LAGP peak and a peak with a maximum at around 54.6 eV, which is close to the binding energy for Li_2_O_2_. The shift to the lower binding energy also indicated that Li_2_CO_3_ was not detected because the Li 1s peak of Li_2_CO_3_ appears higher binding energy of 55.5 eV as shown in Figure S6. Therefore, it is highly probable that the main discharge product in this cell is Li_2_O_2_. Also there is a possibilty that Li_2_CO_3_ is formed by the reaction of Li_2_O_2_ with carbon^28^. However, Li_2_CO_3_ was not detected as described above. Li_2_CO_3_ is formed at the interface between Li_2_O_2_ and carbon and it means that Li_2_CO_3_ is covered with Li_2_O_2_. XPS is surface analytical technique. Therefore, it is considered that Li_2_CO_3_ could not be detected in this measurement, if there is. Then, after charging, the peak intensity in Li 1s region decreased and the peak shifted to the higher energy after charging (Figure S6). The peak after charging can be almost fitted to Li 1s peak of LAGP observed in the spectrum before discharging. It is considered that the discharge products are decomposed during charging. We will elucidate the detailed electrochemical reaction process by using a combination of other spectroscopic measurements in the future. Additionally, analyzing the efficiency of ORR/O_2_ and CO_2_ evolution reactions is important to elucidate the reaction process more accurately. All-solid-state cells have no organic material. Therefore, it is suitable for investigating reaction process related with carbon materials in the air electrode. To analyze the efficiency quantitatively, analysis of consumed and generated gases such as DEMS measurement is effective^7^. It will be also conducted in the future.

In conclusion, the current work demonstrated the potential of the all-solid-state Li-O_2_ battery as a first step. This cell has intrinsically high safety and operated as a Li-O_2_ battery with high theoretical energy density. The cell could operate from room temperature to over 80 °C, a temperature which it is difficult for cells using organic liquid electrolytes. Therefore, this all-solid-state cell extends the operating temperature options for delivering the desired performance. In the present stage, the electrochemical performance, such as rate performance and total capacity of this all-solid-state Li-O_2_ cell, is still insufficient for practical use. However, improvement in cell performance was obtained by reducing the thickness of the solid electrolyte layer. It is presumed that the contact resistance between the lithium anode and the solid electrolyte layer also needs to be improved, as well as that between the solid electrolyte particles and carbon nanotubes in the air electrode. Further optimization of the preparation processes, such as the deposition technique and sintering conditions (temperature, time and atmosphere), will also improve cell performance. In addition, improvements to the air electrode, such as structural control and introduction of a catalyst, are important for enhancing the performance. Thus, the all-solid-state Li-O_2_ cell has significant scope to continue to improve and become practical to use.

## Methods

### Preparation of Li_1+x_Al_y_Ge_2−y_P_3_O_12_ (LAGP) solid electrolyte

LAGP powder for the air electrode and LAGP pellet for the electrolyte layer were prepared using methods reported elsewhere^18^. LAGP was synthesized by the conventional solid-state reaction. Reagent-grade chemicals of Li_2_CO_3_ (Wako Pure Chemical Industries, Ltd., 99%), Al_2_O_3_ (Kojundo Chemical lab. Co., Ltd, 99.999%), GeO_2_ (Kojundo Chemical lab. Co., Ltd, 99.995%) and (NH_4_)H_2_PO_4_ (Wako Pure Chemical Industries, Ltd., 99%) were used as starting materials. The mixture of starting materials was milled at 250 rpm for 4 hours by using a planetary ball mill (Pulverisette 5, Fritsch). The milling process was repeated after heat treatment of 600 °C and 900 °C. The milled precursors were heated at 600 °C for 1 h and 900 °C for 6 h in the oxygen atmosphere. The obtained LAGP powder was used for the air electrode. LAGP pellet for the solid electrolyte layer was prepared from LAGP powder. LAGP powder was pressed into pellets and sintered at 900 °C for 6 h in the oxygen atmosphere. The thickness of the obtained LAGP pellets was about 1 mm. The lithium ion conductivity is about 2 × 10^−4^ S cm^−1^.

### Construction of all-solid-state Li-O_2_ cells

The air electrodes were fabricated on the LAGP pellet by the following procedure. 2 mg of CNT and 40 mg of LAGP powders were mixed in an agate mortar. The mixture was dispersed into 600 mg ethanol solution. A drop of the solution was put on LAGP pellet and dried at room temperature. LAGP pellet with the air electrode was heated at 700 °C for 10 min in an Ar atmosphere. After sintering, air electrode combined with the solid electrolyte layer was obtained as shown in Figure S7. The mass and area of the air electrode were about 0.5 mg and Ø 6 mm. Li anode and Cu current collector were thermally adhered to the reverse side of LAGP pellet with the air electrode and sealed with a plastic film. Al mesh was used as a current collector for the air electrode. The laminate-type cell was sandwiched by two plastic plates and held by a screw clamp. The assembled cell was put into a bottle with connectors for gas flow channels, then the bottle was sealed. All processes were performed in an Ar-filled glove box.

### Evaluation of all-solid-state Li-O_2_ cells

Electrochemical tests were conducted at room temperature, 80 °C, and 120 °C using a charge-discharge measuring device (HJ1001SD8; Hokuto Denko Co.). The discharge and charge current densities were measured in the range from 10 mA g^−1^ to 10 A g^−1^. The current density and cell capacity were normalized by the weight of CNT calculated from the weight of air electrode after sintering and the mixing ratio of LAGP and CNT. Cyclic voltammetry (CV) and electrochemical impedance measurements were performed using a Solartron 1287 coupled with Solartron 1260. The potential sweep was performed at a scan rate of 10 mV s^−1^ at room temperature for the CV measurements. For the impedance measurements, a small perturbation voltage of 10 mV in the frequency range of 1 MHz to 100 mHz was applied.

## Additional Information

**How to cite this article**: Kitaura, H. and Zhou, H. All-solid-state lithium-oxygen battery with high safety in wide ambient temperature range. *Sci. Rep*. **5**, 13271; doi: 10.1038/srep13271 (2015).

## Supplementary Material

Supplementary Information

## References

[b1] Armand M. & Tarascon J.-M. Building better batteries. Nature 451, 652–657 (2008). 18256660 10.1038/451652a

[b2] Abraham K. M. & Jiang Z. A polymer electrolyte-based rechargeable lithium/oxygen battery. J. Electrochem. Soc. 143, 1–5 (1996).

[b3] Zheng J. P., Liang R. Y., Hendrickson M. & Plichta E. J. Theoretical energy density of Li-air batteries. J. Electrochem. Soc. 155, A432–A437 (2008).

[b4] Mizuno F., Nakanishi S., Kotani Y., Yokoishi S. & Iba H. Rechargeable Li-air batteries with carbonate-based liquid electrolytes. Electrochemistry (Tokyo, Jpn.) 78, 403–405 (2010).

[b5] Mizuno F. . Cathode reaction mechanism of non-aqueous Li-O_2_ batteries with highly oxygen radical stable electrolyte solvent. J. Power Sources 228, 47–56 (2013).

[b6] Freunberger S. A. . Reactions in the rechargeable lithium-O_2_ battery with alkyl carbonate electrolytes. J. Am. Chem. Soc. 133, 8040–8047 (2011). 21524112 10.1021/ja2021747

[b7] McCloskey B. D., Bethune D. S., Shelby R. M., Girishkumar G. & Luntz A. C. Solvents’ critical role in nonaqueous lithium-oxygen battery electrochemistry. J. Phys. Chem. Lett. 2, 1161–1166 (2011). 10.1021/jz200352v 26295320

[b8] Jung H.-G., Hassoun J., Park J.-B., Sun Y.-K. & Scrosati B. An improved high-performance lithium-air battery. Nature Chem. 4, 579–585 (2012). 22717445 10.1038/nchem.1376

[b9] Adams B. D. . Towards a stable organic electrolyte for the lithium oxygen battery. Adv. Energy Mater. 5, 1400867 (2015).

[b10] Schneider A. A., Harney D. E. & Harney M. J. The lithium-iodine cell for medical and commercial applications. J. Power Sources 5, 15–23 (1980).

[b11] Liang C. C. Conduction characteristics of the lithium iodide-aluminum oxide solid electrolytes. J. Electrochem. Soc. 120, 1289–1292 (1973).

[b12] Bates J. B., Dudney N. J., Neudecker B. J., Ueda A. & Evans C. D. Thin-film lithium and lithium-ion batteries. Solid State Ionics 135, 33–45 (2000).

[b13] Julien C., Saikh S. & Balkanski M. Electrochemical properties and cycling performance of composite electrodes in solid state lithium batteries. Mater. Sci. Eng. B14, 121–126 (1992).

[b14] Tatsumisago M. & Hayashi A. Preparation of lithium ion conducting glasses and glass-ceramics for all-solid-state batteries. J. Non-Cryst. Solids 354, 1411–1417 (2008).

[b15] Knauth P. Inorganic solid Li ion conductors: An overview. Solid State Ionics 180, 911–916 (2009).

[b16] Kamaya N. . A lithium superionic conductor. Nature Mater. 10, 682–686 (2011). 21804556 10.1038/nmat3066

[b17] Kumar B. . A solid-state, rechargeable, long cycle life lithium-air battery. J. Electrochem. Soc. 157, A50–A54 (2010).

[b18] Kitaura H. & Zhou H. Electrochemical performance and reaction mechanism of all-solid-state lithium-air batteries composed of lithium, Li_1+x_Al_y_Ge_2*−*y_(PO_4_)_3_ solid electrolyte and carbon nanotube air electrode. Energy Environ. Sci. 5, 9077–9084 (2012).

[b19] Ogasawara T., Débart A., Holzapfel M., Novák P. & Bruce P. G. Rechargeable Li_2_O_2_ electrode for lithium batteries. J. Am. Chem. Soc. 128, 1390–1393 (2006). 16433559 10.1021/ja056811q

[b20] Park J.-B. . Influence of temperature on lithium-oxygen battery behavior. Nano Lett. 13, 2971–2975 (2013). 23679097 10.1021/nl401439b

[b21] Laoire C. Ó., Mukerjee S., Plichta E. J., Hendrickson M. A. & Abraham K. M. Rechargeable lithium/TEGDME-LiPF_6_/O_2_ battery. J. Electrochem. Soc. 158, A302–A308 (2011).

[b22] Lim H.-K. . Toward a lithium-“air” battery: The effect of CO_2_ on the chemistry of a lithium-oxygen cell. J. Am. Chem. Soc. 135, 9733–9742 (2013). 23758262 10.1021/ja4016765

[b23] Murugan R., Thangadurai V. & Weppner W. Fast lithium ion conduction in garnet-type Li_7_La_3_Zr_2_O_12_ Angew. Chem. Int. Ed. 46, 7778 (2007). 10.1002/anie.200701144 17803180

[b24] Nakayam M., Kotobuki M., Munakata H., Nogami M. & Kanamura K. First-principles density functional calculation of electrochemical stability of fast Li ion conducting garnet-type oxides. Phys. Chem. Chem. Phys. 14, 10008 (2012). 22711381 10.1039/c2cp40634a

[b25] Hartmann P. . Degradation of NASICON-type materials in contact with lithium metal: Formation of mixed conducting interphases (MCI) on solid electrolytes. J. Phys. Chem. C 117, 21064–21074 (2013).

[b26] Kobayashi E., Plashnitsa L. S., Doi T., Okada S. & Yamaki J. Electrochemical properties of Li symmetric solid-state cell with NASICON-type solid electrolyte and electrodes. Electrochem. Commun. 12, 894–896 (2010).

[b27] Ohta S. . All-solid-state lithium ion battery using garnet-type oxide and Li_3_BO_3_ solid electrolytes fabricated by screen-printing. J. Power Sources 238, 53–56 (2013).

[b28] McCloskey B. D. . Twin problems of interfacial carbonate formation in nonaqueous Li-O_2_ batteries. J. Phys. Chem. Lett. 3, 997–1001 (2012). 10.1021/jz300243r 26286562

[b29] Hu Y. . Size effect of lithium peroxide on charging performance of Li-O_2_ batteries. Nanoscale 5, 177 (2014). 24219997 10.1039/c3nr04728h

[b30] Lim H.-D. . Enhanced power and rechargeability of a Li-O_2_ battery based on a hierarchical-fibril CNT electrode. Adv. Mater. 25, 1348–1352 (2013). 23255225 10.1002/adma.201204018

[b31] Li J., Zhou G., Zhang Z., Lai Y. & Jia M. Partially cracked carbon nanotubes as cathode materials for lithium-air batteries. ECS Electrochem. Lett. 2, A25–A27 (2013).

[b32] Kitaura H. & Zhou H. Electrochemical performance of solid-state lithium-air batteries using carbon nanotube catalyst in the air electrode. Adv. Energy Mater. 2, 889–894 (2012).

[b33] Peng Z. . Oxygen reactions in a non-aqueous Li^+^ Electrolyte. Angew. Chem. Int. Ed. 50, 6351–6355 (2011). 10.1002/anie.201100879 21604342

[b34] Yao K. P. C. . Thermal stability of Li_2_O_2_ and Li_2_O for Li-air batteries: *In situ* XRD and XPS studies. J. Electrochem. Soc. 160, A824–A831 (2013).

[b35] Lu Y.-C. . *In situ* ambient pressure X-ray photoelectron spectroscopy studies of lithium-oxygen redox reactions. Sci. Rep. 2, #715 (2012). 10.1038/srep00715 PMC3465812 23056907

