# Supplementary material for: All-solid-state lithium-oxygen battery with high safety in wide ambient temperature range
Source: Sci Rep. 2015 Aug 21;5:13271. doi: 10.1038/srep13271 (PMC4543982; doi:10.1038/srep13271)
Supplement: Supplementary Information [file srep13271-s1.doc]

Supplementary Information for

**All-solid-state lithium-oxygen battery with high safety in wide ambient temperature range**

Hirokazu Kitaura, Haoshen Zhou*

**Corresponding author:**

Haoshen Zhou

Energy Technology Research Institute, Advanced Industrial Science and Technology,

Tsukuba Central 2, 1-1-1, Umezono, Tsukuba, Ibaraki 305-8568, JAPAN.

Tel.: +81-29-8615795, Fax.: +81-29-8613489

E-mail address: hs.zhou@aist.go.jp.

**Supplementary Figures**

**Supplementary Figure S1 Rate performance of all-solid-state Li-O_2_ cell under the current densities changed in a step by step manner.** (a) Discharge-charge curves for cell using LAGP pellet with the thickness of about 1 mm under current densities changed from 10 to 1000 mA g^-1^ at room temperature and (b) enlarged view of initial data.

**Supplementary Figure S2 Rate performance of all-solid-state Li-O_2_ cell under the current densities changed in a step by step manner.** (a) Discharge-charge curves for cell using LAGP pellet with the thickness of about 0.5 mm under current densities changed from 10 to 5000 mA g^-1^ at room temperature and (b) enlarged view of initial data.

**Supplementary Figure S3 Rate performance of all-solid-state Li-O_2_ cell under the current densities changed in a step by step manner.** (a) Discharge-charge curves for cell using LAGP pellet with the thickness of about 1 mm under current densities changed from 10 to 10000 mA g^-1^ at 80 ^o^C and (b) enlarged view of initial data.

**Supplementary Figure S4 XPS spectra of LAGP-CNT cathodes before (black) and after discharge (red) in Li 1s, O 1s, C 1s, P 2p, Ge 3d and Al 2p regions calibrated by using Ge 3d main peak (32.3 eV) of Ge^4+^.**

**Supplementary Figure S5 XPS spectra of LAGP-CNT cathodes before (black) and after discharge (red) in C 1s region calibrated by using 284.5 eV of C-C or CH_x_ binding energy.**


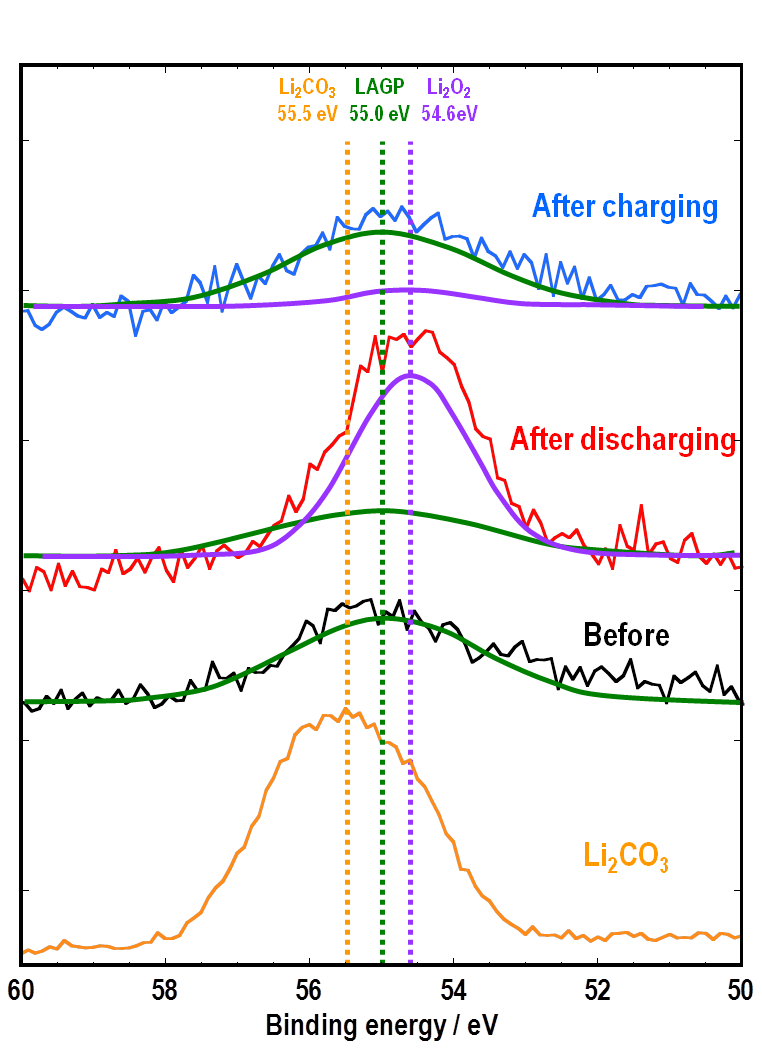


**Supplementary Figure S6 XPS spectra of LAGP-CNT cathodes before (black), after discharge (red) and after charge (blue) in Li 1s region calibrated by using Ge 3d main peak (32.3 eV) of Ge^4+^ and Li_2_CO_3_ reagent in Li 1s region calibrated by using C 1s peak related with carbonate (290.0 eV).**

**XPS analysis** The XPS measurements were carried out at a Ulvac-Phi XPS-1800 spectrometer. Monochromatic Al-Kα excitation (350 W) and a low-energy electron charge neutralizer were used. The air electrode powder was scratched from the LAGP cell and fixed on Cu tape in the glove box. The samples were transferred into the XPS chamber by using glove bag flowing nitrogen gas. The spectra in Fig. S4 and S6 were calibrated by using Ge 3d main peak (32.3 eV) of Ge^4+^. The peak positions of Li 1s, P 2p, and Al 2p before discharge are consistent with those of LAGP [21]. Also, the peak positions of P 2p and Al 2p after discharge are consistent with those before discharge and it indicates that the spectral calibration by Ge 3d is valid. On the other hand, the peak positions of C 1s both before and after discharge are inappropriate because of the inhomogeneous charge effect owing to the different conductivity between LAGP and carbon species [Shabtai *et al*., *J. Am. Chem. Soc.*, 2000, **122**, 4959]. We compared to these spectra with the spectra of Li_2_CO_3_ reagent and mixture of CNT and Li_2_CO_3_ reagent to determine the peak for the correct calibration. In conclusion, the second lowest peak of C 1s spectra were calibrated to 284.5 eV as C-C or CH_x_ binding energy (Fig. S5). The shift of peak top in the Li 1s region after discharge in Fig. S4 is due to the formation of the lithium compound and the predictable lithium compounds are insulative as well as LAGP. Therefore, it is presumed that Li 1s spectrum after discharge, which contains both peaks of the insultative LAGP and the insulative produced lithium compound, can be calibrated by using the Ge^4+^ peak of LAGP.


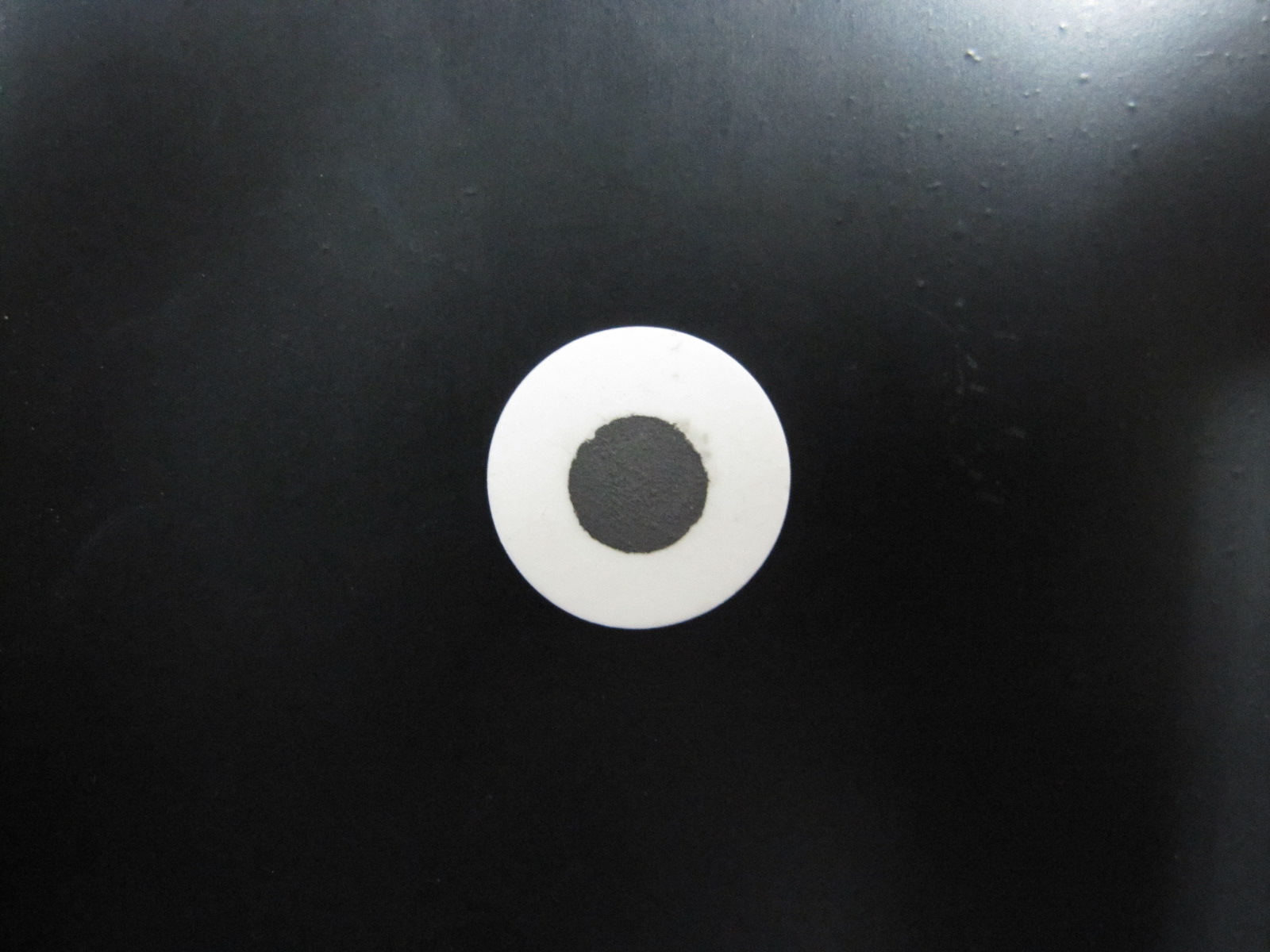


**Supplementary Figure S7 Photograph of LAGP-CNT cathode on LAGP pellet after heat treatment. Black circle area is cathode and white area is LAGP only.**
